# Modelling the transmission and persistence of African swine fever in wild boar in contrasting European scenarios

**DOI:** 10.1038/s41598-020-62736-y

**Published:** 2020-04-03

**Authors:** Xander O’Neill, Andy White, Francisco Ruiz-Fons, Christian Gortázar

**Affiliations:** 1 Maxwell Institute for Mathematical Sciences, Department of Mathematics, Heriot-Watt University, Edinburgh, EH14 4AS UK; 2 SaBio, Instituto de Investigación en Recursos Cinegéticos IREC (UCLM & CSIC), 13005 Ciudad Real, Spain

**Keywords:** Differential equations, Numerical simulations, Applied mathematics

## Abstract

African swine fever (ASF) is a severe viral disease that is currently spreading among domestic pigs and wild boar (*Sus scrofa*) in large areas of Eurasia. Wild boar play a key role in the spread of ASF, yet despite their significance, little is known about the key mechanisms that drive infection transmission and disease persistence. A mathematical model of the wild boar ASF system is developed that captures the observed drop in population density, the peak in infected density and the persistence of the virus observed in ASF outbreaks. The model results provide insight into the key processes that drive the ASF dynamics and show that environmental transmission is a key mechanism determining the severity of an infectious outbreak and that direct frequency dependent transmission and transmission from individuals that survive initial ASF infection but eventually succumb to the disease are key for the long-term persistence of the virus. By considering scenarios representative of Estonia and Spain we show that faster degradation of carcasses in Spain, due to elevated temperature and abundant obligate scavengers, may reduce the severity of the infectious outbreak. Our results also suggest that the higher underlying host density and longer breeding season associated with supplementary feeding leads to a more pronounced epidemic outbreak and persistence of the disease in the long-term. The model is used to assess disease control measures and suggests that a combination of culling and infected carcass removal is the most effective method to eradicate the virus without also eradicating the host population, and that early implementation of these control measures will reduce infection levels whilst maintaining a higher host population density and in some situations prevent ASF from establishing in a population.

## Introduction

African swine fever (ASF) virus, belonging to the Asfarviridae family, is a virulent virus affecting domestic pigs, wild boar (*Sus scrofa*) and African wild suids. Infection from highly virulent strains leads to near 100% mortality at the individual level^1–4^, and outbreaks typically lead to significant population loss^5^. ASF has a large economic impact in affected countries due to losses in pig production and food security threats and is listed as a notifiable disease by the World Organisation for Animal Health^4,6^. Gaining a clear understanding of the infection and transmission dynamics and developing strategies to control ASF outbreaks is a key, current priority^7^.

African swine fever is endemic in wild suids and domestic pigs in sub-Saharan Africa. The virus first emerged in Europe in 1957 in Portugal, spreading subsequently to Spain, France, Belgium, the Netherlands, Italy and Malta. The epidemic was eradicated in mainland Europe and Malta in 1999 through control of infected and at-risk domestic pig populations and by imposing strict bio-security measures^8^. However, the infection still persists on Sardinia^9,10^. A second epidemic started in Georgia in 2007. In the same year, ASF was reported in wild boar in the Russian Federation and by 2014 it had spread to Ukraine, Belarus, Estonia, Latvia, Lithuania and Poland^5^, where ASF remains present. Recent outbreaks have been reported in the Czech Republic (the only successfully eradicated local outbreak), Belgium (ongoing local outbreak), Slovakia and several south-east European countries^11^. This suggests ASF is still spreading and therefore poses a substantial threat to some of Europe’s most significant pig farming regions. The presence of ASF infection in wild boar populations represents a current and significant disease management challenge across Eurasia^7^. The challenge is intensified as the density of wild boar has experienced an unprecedented increase over the last decades^12^, which is likely to enhance their ability to contribute to ASF spread and maintenance.

Despite the documented importance of wild boar in the spread of ASF, an understanding of the epidemiological dynamics is incomplete and little is known about the key mechanisms that drive infection transmission and disease persistence^13,14^. ASF typically leads to an acute infection with close to 100% individual mortality around 4–9 days following exposure^1,15^. A less common form of infection is possible, where individuals do not die but develop a persistent infection, which may be accompanied by signs of sub-acute or chronic disease^13,14^. This invariably leads to death, with the potential to excrete virus in association with the resurgence of viraemia^14,16^. Such individuals are termed type 1 ‘survivors’ by Ståhl *et al*.^14^ since they survive the initial ASF infection and we will call such individuals ‘survivors’ in this study. Infection can lead to a population reduction of 85–95% in the initial epidemic phase^5^. The disease does not exhibit a typical epidemic pattern for highly virulent, acute infections with self-limiting localized epidemics^15^, and instead can persist in low density populations at low prevalence (1–3%) for several years following an epidemic^5^. ASF is highly contagious and potential transmission routes include direct transmission through close contact with infected individuals^17^ - which may occur within social groups and more widely if social groups congregate at feeding stations or water holes. Transmission may also occur via environmental contamination due to the persistence of infection in carcasses. Such carcasses can be contaminated with ASF virus for a considerable length of time^18^, and contact with carcasses poses a significant risk of transmission^13,15^. Understanding these key infection processes is critical to predicting the effectiveness of different control strategies, such as culling and carcass removal, to manage and eradicate ASF^7,16^.

Mathematical models have played a key role in understanding the processes that drive epidemiological dynamics in wildlife populations^19^,^20^. In the context of ASF, statistical spatial models have shown that survivor individuals and infectious residue from dead animals may be important for the spread and persistence of ASF^21^. Statistical models have also been fitted to data to examine the spread of ASF in the Russian Federation^2^,^4^, the impact of control zones on the (hypothetical) persistence of ASF in Denmark^22^, and to examine the between farm spread of ASF in domestic pigs in Sardinia^23^, suggesting wild boar management may be a key component in reducing spread in farmed populations. Model studies have also been used to undertake a risk assessment for the spread of ASF. These studies have used statistical data fitting approaches to determine the risk of ASF introduction through contaminated pork products^24,25^, have linked ASF infection data to meteorological records to make global predictions of ASF outbreaks^26^, and have identified risk factor indicators to predict the spread of ASF in Europe^27,28^, with wild boar density classified as a key indicator. These model approaches have not focused on determining the underlying epidemiological processes responsible for infection. A stochastic model (based on a process based deterministic model), that focused on direct transmission of infection, was developed to assess the impact of the implementation of different disease control strategies^6^. Here the focus was not on understanding the importance of the key epidemiological processes but on assessing the effectiveness of a hypothetical vaccine and of bio-security control measures, modelled implicitly as a reduction in infection transmission. They showed that these strategies could reduce population mortality due to ASF, particularly if the control measures were applied soon after the initial outbreak^6^.

In this study we develop a deterministic model of ASF in wild boar to uncover the key transmission and infection maintenance processes. The model includes direct transmission and transmission from infected carcasses and the possibility of survivor individuals, all of which have been implicated in ASF outbreaks and persistence^14–16,18^. We compare the model results to key observed criteria for the epidemiological dynamics of ASF in Estonia to determine the important processes that drive the infection and to fit model parameters. The fitted model will then be used to test the effectiveness of disease management by explicitly including the impact of culling and carcass removal. We will also consider a model set-up that represents conditions in Spain, where wild boar densities are considerably higher than in Estonia and where the persistence of carcasses is reduced due to climatic conditions and the presence of abundant obligate scavengers, such as vultures. The results will highlight the usefulness of mathematical modelling for disease management and provide important new information to understand the epidemiological dynamics of ASF and to inform policy to control its spread.

## Methodology

Detailed data on the local, temporal population and epidemiological dynamics for ASF are not available. Instead we use information from published studies to develop and fit a model to key epidemiological criteria observed for ASF in wild boar in eastern Europe. These criteria are:

1. An 85–95% drop in total population density after an epidemic^5^,^29^.
2. A peak in the number of infected individuals approximately 6 months after the virus is initially discovered^5^. In the model results this criterion is specified as a peak in infected density in a 4–10 month period following the initial infection.
3. The persistence of the virus several years after the initial epidemic^5^. In the model results this criterion is specified as having a 1–3% prevalence 3 years after the initial epidemic.

### Mathematical Modelling

We develop a model for the temporal dynamics of ASF in wild boar which is an extension of classical, compartmental disease modelling frameworks^20,30^, and of models for disease transmission in wild boar^31,32^. We consider a model that separates wild boar into two age classes: piglets (hosts aged 0 − 10 months on average, subscript *P*) and yearlings/adults (known hereafter as adults, subscript *A*). We do this as there are key differences in reproduction, natural mortality and mortality due to hunting between these age-classes. We further classify the population in terms of the key infection status of individuals. This is through the classes *S*, uninfected and susceptible to infection; *I*, infected and able to transmit the virus; *C*, survivor individuals which do not transmit the virus but can revert to the infected (*I*) class and *D*, infected carcasses which can transmit the virus. The model is detailed below:

1 $$\begin{array}{lll}\frac{d{S}_{P}}{dt} & = & a(t)A-{\beta }_{F}\frac{{S}_{P}}{N}I-{\beta }_{E}{S}_{P}D-\alpha {S}_{P}-{b}_{P}{S}_{P}-{b}_{C}{S}_{P},\\ \frac{d{S}_{A}}{dt} & = & -{\beta }_{F}\frac{{S}_{A}}{N}I-{\beta }_{E}{S}_{A}D+\alpha {S}_{P}-{b}_{A}{S}_{A}-{b}_{H}{S}_{A}-{b}_{C}{S}_{A},\\ \frac{d{I}_{P}}{dt} & = & {\beta }_{F}\frac{{S}_{P}}{N}I+{\beta }_{E}{S}_{P}D-\alpha {I}_{P}-\gamma {I}_{P}+\kappa {C}_{P}-{b}_{P}{I}_{P}-{b}_{C}{I}_{P},\\ \frac{d{I}_{A}}{dt} & = & {\beta }_{F}\frac{{S}_{A}}{N}I+{\beta }_{E}{S}_{A}D+\alpha {I}_{P}-\gamma {I}_{A}+\kappa {C}_{A}-{b}_{A}{I}_{A}-{b}_{H}{I}_{A}-{b}_{C}{I}_{A},\\ \frac{d{C}_{P}}{dt} & = & \gamma (1-\rho ){I}_{P}-\kappa {C}_{P}-\alpha {C}_{P}-{b}_{P}{C}_{P}-{b}_{C}{C}_{P},\\ \frac{d{C}_{A}}{dt} & = & \gamma (1-\rho ){I}_{A}-\kappa {C}_{A}+\alpha {C}_{P}-{b}_{A}{C}_{A}-{b}_{H}{C}_{A}-{b}_{C}{C}_{A},\\ \frac{dD}{dt} & = & \gamma \rho I+{b}_{P}{I}_{P}+{b}_{A}{I}_{A}-dD-rD.\end{array}$$

Here, *I* = *I*_*P*_ + *I*_*A*_ denotes the total infected population, *A* = *S*_*A*_ + *I*_*A*_ + *C*_*A*_ denotes the total adult population and *N* = *S*_*P*_ + *S*_*A*_ + *I*_*P*_ + *I*_*A*_ + *C*_*P*_ + *C*_*A*_ denotes the total population of (living) wild boar. We base our model on the system in Estonia and parameterise the model where possible from published data. Adults give birth to susceptible piglets during a defined breeding season with seasonal birth rate *a*(*t*). We consider two forms for *a*(*t*), a ‘natural’ population and a population which receives supplementary feeding (see Fig. S1 in the Supplementary Information). The maturation of piglets to adults occurs at rate *α* = 12/10 which reflects an average maturation time of 10 months^33^. In the absence of the disease wild boar suffer mortality at rate *b*_*P*_, *b*_*A*_ and *b*_*H*_ representing natural death of piglets and adults and mortality due to hunting respectively. For Estonia the total annual adult mortality is 53%, of which 93% is due to hunting and 7% due to other forms of mortality^34^ giving *b*_*H*_ = 0.69 and *b*_*A*_ = 0.05. Piglet annual mortality increases from 50% at low density to 95% at high density due to crowding effects and is given by *b*_*P*_ = (*b*_0_ + *b*_1_*N*) where ${b}_{0}=\log \,(2)$ and ${b}_{1}=\sigma \frac{(\bar{a(t)}-{b}_{A}-{b}_{H})\alpha -{b}_{0}({b}_{A}+{b}_{H})}{({b}_{A}+{b}_{H})K}$ where $\bar{a(t)}$ represents the average annual birth rate, *K* the carrying capacity, defined as the average annual population size in the absence of ASF, and *σ* denotes a scaling term to ensure that the average population density equals the carrying capacity. We assume a carrying capacity, *K*, of 2 *km*^−2^ in natural populations and 4 *km*^−2^ under supplementary feeding. To allow consideration of disease control measures, we include culling, at rate *b*_*C*_ and the removal of carcasses, at rate *r*.

For the infection dynamics, a susceptible can become infected due to direct contact with an infected individual via frequency dependent transmission, *β*_*F*_ or due to environmental transmission through contact with an infected carcass, *β*_*E*_. A proportion, *ρ*, of infected individuals suffer disease induced mortality at rate *γ* = 365/5, reflecting an average lifespan of 5 days for an individual with ASF^1^. A proportion 1 − *ρ* of infected can enter the survivor class, which does not incur disease induced mortality. Survivors can revert back to the infected class (where they incur disease-induced mortality) at rate *κ* = 12/6 implying that the average time spent in the survivor class is 6 months^1^. Individuals that die whilst infected are classed as infected carcasses and can transmit infection for on average 8 weeks^18,35^, giving a decay rate of carcasses *d* = 52/8. Note, we also examine the impact of a range of average times spent in the survivor class and a range of carcass degradation rates.

To initiate the model we introduce a low level of infection (*I* is set to 0.2% of the carrying capacity, *K*) to a population at its carrying capacity. We make no assumptions about the source of the initial infection, which could include contact with an infected individual from a neighbouring population or human-mediated introduction. We assume the model (Eq. (1)) includes the key mechanisms responsible for infection transmission within a population following the initial infection and undertake an exploration in parameter space to determine the combinations of transmission terms (*β*_*E*_ and *β*_*F*_) and the proportion of survivors (1 − *ρ*) that can satisfy the epidemiological criteria for ASF outlined in this study. We aim to interpret the results in terms of the epidemiological mechanisms responsible for ASF dynamics and maintenance, examine the epidemiological consequences of these results and test the impact of disease control measures in the form of increased carcass removal and culling. We also examine the potential impact of ASF and disease control in other regions that may have higher density populations of wild boar (i.e. Spain), higher populations of obligate scavengers and higher temperatures which significantly contribute to faster carcass degradation and virus decay compared to Estonia. We adjust the model to represent the situation in Spain by considering carrying capacities of 5 *km*^−2^ in natural populations and 10 *km*^−2^ under supplementary feeding^36^. Total annual adult mortality is now 56%, of which 60% is due to hunting and 40% due to other forms of mortality^37^. This gives *b*_*H*_ = 0.49 and *b*_*A*_ = 0.33. To represent increased temperature and increased activity from obligate scavengers (e.g. vultures)^38^, we assume an individual infected carcass can transmit infection for on average one week (giving *d* = 52).

## The drivers for the epidemiological dynamics of ASF

**Figure 1:**
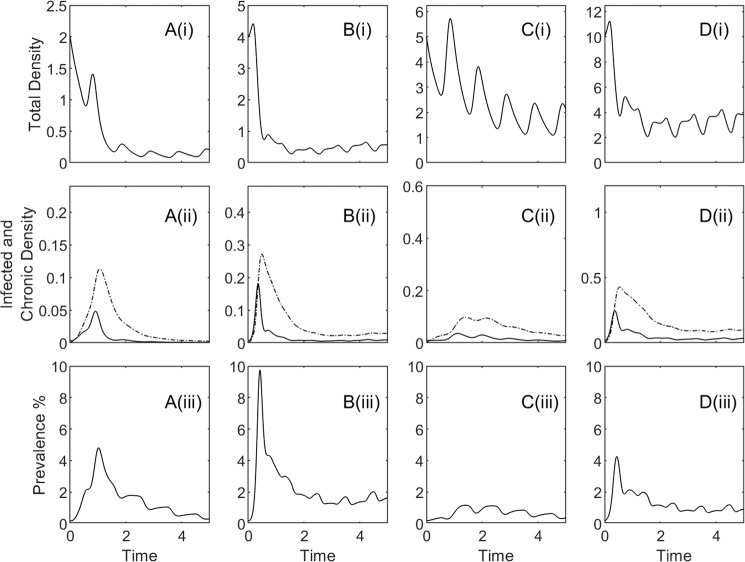
Population densities and prevalence over time for the model described by equations (1). All results were obtained using MATLAB software, specifically the built-in ODE solver packages. Total densities are given in (i), infected (solid line) and survivor (dashed line) densities in (ii), with prevalence, defined as *I*/*N*, in (iii). The plots in A and B represent the scenario for Estonia, under natural conditions and with supplementary feeding respectively. In C and D we show the model results for the scenario that represents Spain, under natural conditions and with supplementary feeding respectively. For Estonia we have *d* = 52/8, with *K* = 2 (**A**) and *K* = 4 (**B**). For Spain *d* = 52 with *K* = 5 (**C**) and *K* = 10 (**D**). Other parameters are *β*_*F*_ = 63, *β*_*E*_ = 2, *ρ* = 0.85, *b*_*C*_ = 0 and *r* = 0 (see also section S1).

We undertake a sensitivity analysis to determine the range of parameters that produce model outputs satisfying the epidemiological criteria observed for ASF and outlined in this study (further details of the sensitivity to model parameters are provided in the supplementary information, section S2). A key finding is that frequency dependent transmission, environmental transmission and the progression from an infected state to the survivor state are required to satisfy the criteria for ASF (Fig. S2). Importantly, this suggests that survivor individuals may play a key role in the long-term persistence of ASF. For parameters that satisfy the criteria the epidemiological dynamics are similar and shown for representative parameters in Fig. 1A. This highlights the crash in total host density (Fig. 1Ai), the epidemic outbreak in infected hosts and consequently in survivor individuals (Fig. 1Aii) and a peak and persistence of low prevalence infection following the outbreak (Fig. 1Aiii). While there are more acute (infected) cases than survivor cases over the course of the epidemic, the density of the infected class (*I*) may be lower than the density of the survivor class (*C*) due to the high rate of disease induced mortality for *I* (which results in infected carcasses *D*). The epidemiological dynamics arise from the following processes:

1. Density dependent environmental transmission is the key process driving the initial population crash. Model results when environmental transmission is excluded (Fig. S3A) show no epidemic outbreak, and as a result no drop in population density. When environmental transmission is high (Fig. S3C) the crash in population density is severe and drops below the level defined by our epidemiological criteria.
2. Frequency dependent transmission and the progression and subsequent reversion from survivor to infected individuals allows the infection to be sustained at low density. Without survivor individuals the disease is self-limiting and fades out after the epidemic (Fig. S5), whilst with low frequency dependent transmission the disease does not persist in the long-term at low population density (Fig. S4). If frequency dependent transmission is high the population exhibits pathogen driven extinction (Fig. S4C) and if a high proportion of infected individuals survive the initial infection and enter the survivor class the population does not exhibit the required reduction in density.

In our model study survivor individuals revert back to the infected state after an average of six months, but there is uncertainty in this duration^39^. We use the model to show that the epidemiological criteria for ASF can be satisfied for a range of durations in the survivor class (from 2 months to 9 months, see Fig. S6). In order to satisfy the epidemiological criteria when the length of time in the survivor class decreases, the proportion of individuals that survive the infection must increase, leading to an increase in the density of survivor individuals.

## The dynamics of ASF in different regions

The model sensitivity analysis was undertaken to match observed epidemiological dynamics for natural populations in Estonia and provide a default set of infection parameters. We use these parameters to examine the impact of ASF for additional scenarios in Estonia that simulate when wild boar are supplementary fed (Fig. 1B) and for scenarios representative of Spain for natural (Fig. 1C) and supplementary fed (Fig. 1D) populations.

In Estonia (with both natural conditions and supplementary feeding) and in Spain under supplementary feeding there is an initial epidemic outbreak. This causes an 85–95% population reduction in Estonia (Fig. 1A(i),B(i)) and a 60–70% reduction in Spain (Fig. 1D(i)). In Spain, under natural conditions, the infectious outbreak develops more slowly and the drop in population density is more gradual compared to the other scenarios. Nevertheless, the infection still leads to a 60–70% reduction in population density (Fig. 1C(i)). In all scenarios the infection persists at low prevalence for several years after the initial infectious outbreak (Fig. 1). Under natural conditions the disease persistence has a low but declining prevalence of infection (Fig. 1A,C), but with the inclusion of supplementary feeding the infection persists with a low and stable level of prevalence (Fig. 1B,D). The addition of feeding also leads to a more severe infectious outbreak with a rapid initial drop in population density. In the long-term, the disease is likely to fade out in the absence of feeding and persist at low prevalence with the inclusion of feeding, where the population is likely to be regulated by the disease.

## The impact of different control methods

**Figure 2:**
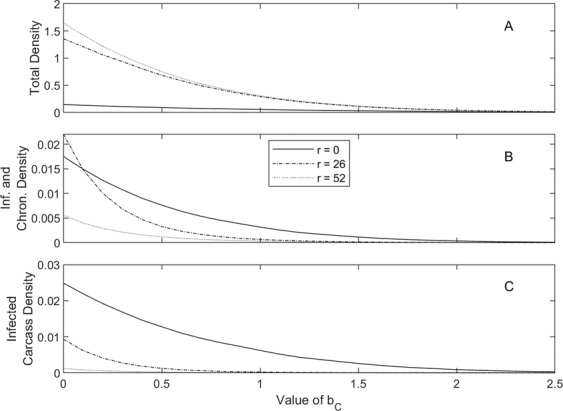
Population response to a varying culling intensity, *b*_*C*_, with three different carcass removal rates *r* = 0 (solid line), *r* = 26 (dashed) and *r* = 52 (dotted), for the model represented by equations (1). The total density, *N*, is given in A, with infected and survivor density in B and carcass density in C. Results are shown for the scenario that represents natural conditions in Estonia (see Fig. 1 for parameters) and show the average densities between the years 2 and 3 following disease introduction. Control measures were implemented as soon as the virus is first discovered, defined as the time when carcass levels first reach a density of 0.02. The value *b*_*C*_ corresponds to a culling proportion equal to $1-{e}^{-{b}_{C}}$, per year, of the total population.

**Figure 3:**
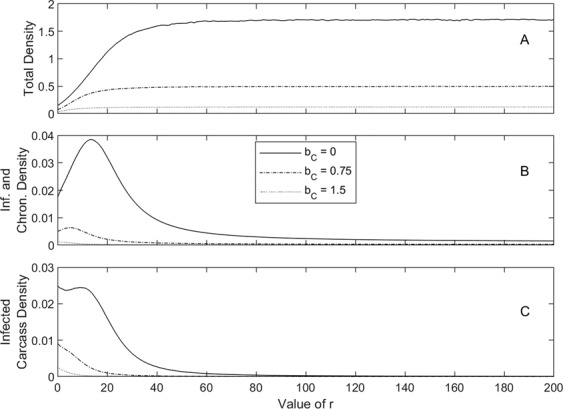
Population response to a varying carcass removal rate, *r*, with three different culling intensities *b*_*C*_ = 0 (solid line), *b*_*C*_ = 0.75 (dashed) and *b*_*C*_ = 1.5 (dotted), for the model represented by equations (1). The total density, *N*, is given in A, with infected and survivor density in B and carcass density in C. Results are shown for the scenario that represents natural conditions in Estonia (see Fig. 1 for parameters) and show the average densities between the years 2 and 3 following disease introduction. Control measures were implemented as soon as the virus is first discovered, defined as the time when carcass levels first reach a density of 0.02. The value *r* corresponds to an average removal time, in years, of 1/*r*.

We consider two control methods: culling and the removal of infected carcasses, and initially focus on the scenario in Estonia under natural conditions. When culling alone is used (Fig. 2, with *r* = 0) an increase in the rate of culling reduces the total density as well as the infected, survivor and infected carcass densities. The disease persists at low population density indicating that culling may not be effective at eradicating the disease without also eradicating the population. When carcass removal alone is used (Fig. 3, with *b*_*C*_ = 0), we see an increase in total population density as the carcass removal rate is increased. This occurs as the removal of carcasses reduces environmental transmission and so reduces the population level mortality due to ASF. At low levels of carcass removal, the increase in total population supports an increase in the number of infected individuals but as the carcass removal rate increases further the infected level peaks and then decreases. Note that high levels of carcass removal can eliminate infected carcasses yet ASF is still supported at low density through frequency dependent transmission and survivor individuals. While the impact on population density is low it emphasizes that carcass removal alone may not be sufficient to eradicate ASF. When we consider a combination of disease control methods (Figs. 2 & 3) model results indicate that it is possible to eradicate all sources of infection without eradicating the host population. In particular, varying the culling rate for fixed levels of carcass removal show a clear difference in the culling threshold for disease eradication and population eradication (Fig. 2). Varying the carcass removal rate for fixed levels of culling shows that the disease can be eradicated while maintaining a positive host population level (Fig. 3). The impacts of these disease control methods show similar trends for the scenarios that represent Estonia with feeding and for parameters representative of Spain (see Figs. S7, S8, S11, S12, S15 & S16). However, the elevated rate of natural carcass removal in Spain means that the infection can be eradicated through culling only and more generally this makes the virus easier to control in Spain compared to Estonia. Nonetheless, in all scenarios the removal of carcasses (applied when the infection is detected) cannot eradicate the disease, although a high level of removal does reduce the level of infection and impact on host population density. Our model results suggest that a combination of control procedures will be the most effective method to eradicate the disease while maintaining a viable population of wild boar.

**Figure 4:**
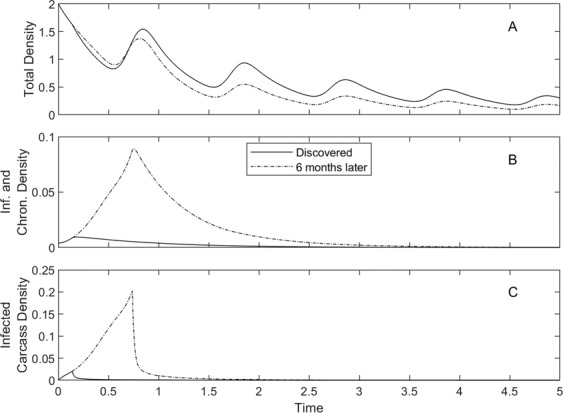
Population response to the combination of culling, at fixed rate *b*_*C*_ = 0.75, and carcass removal, at fixed rate *r* = 52, for the model represented by equations (1) and for parameters that represent the scenario in Estonia under natural conditions (see Fig. 1 for parameters).The total density, *N*, is given in A, with infected and survivor densities in B and prevalence in C. The results are shown for two different control implementation times: when the virus is first discovered (solid line), and six months after the virus was discovered (dashed line).

**Figure 5:**
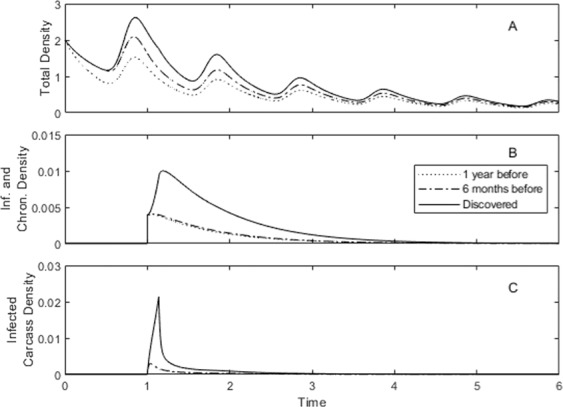
Population response to the combination of culling, at fixed rate *b*_*C*_ = 0.75, and carcass removal, at fixed rate *r* = 52, for the model represented by equations (1) and for parameters that represent the scenario in Estonia under natural conditions (see Fig. 1 for parameters).The total density, *N*, is given in A, with infected and survivor densities in B and prevalence in C. The results are shown for three different control implementation times: one year before the onset of the virus (dotted line), six months before the onset of the virus (dashed line), and when the virus is first discovered (solid line).

We also test the impact of the timing of the implementation of control (Fig. 4). Applying control as soon as the disease is discovered in a population is significantly more effective than if control is applied later, when the infection is at epidemic levels. In particular, the early application of control reduces the level of infected individuals while maintaining a higher host population density. This reduction in the level of infected individuals is further enhanced if control is applied prior to the introduction of infection (Fig. 5) and these lower levels of infection are likely to reduce the risk of infectious spread to other populations. In the general control methods reduce the population density and the rate of transmission to a level that cannot support ASF. This trend is seen across the different scenarios considered in this study (Figs. S9, S13 and S17). It should be noted however, for Spain, where there is assumed to be high levels of ‘natural’ carcass degradation, that high levels of culling can reduce the host density to a level that cannot support the disease and could prevent ASF from establishing if applied prior to an infectious outbreak (see Figs. S11 and S15 with r = 0).

## Discussion

There is limited information regarding the key processes responsible for ASF disease transmission and the mechanisms that allow the virus to persist at low density in wild boar populations whilst maintaining a high disease induced mortality rate. We have developed an age-structured mathematical model that includes a range of potential transmission mechanisms with the aim of understanding the epidemiological dynamics of ASF in wild boar. The model captures the observed drop in population density^5,29^, the peak in infected density^5^ and the persistence of the virus^5^ and gives an insight into the key processes that drive these dynamics.

Our model results indicate that environmental transmission from infected carcasses to susceptible individuals and frequency dependent transmission from infected to susceptible individuals are key factors in producing a disease outbreak and the persistence of the disease at low population levels, respectively. These mechanisms have been highlighted as key factors in driving ASF dynamics in warthogs^13,17^, with persistence linked to the long-term survival of the virus in the environment^15,18,40^. Gallardo *et al*.^39^ suggests that sub-clinically infected, chronically infected or survivor pigs are likely to play an important role in disease persistence in endemic areas and in sporadic outbreaks or ASF introduction into disease-free zones. Our model analysis clarifies the potential role of hosts that can survive the initial ASF infection but that may revert back to an infected state indicating that they are necessary for the persistence of ASF in low density host populations. A key message is that all three routes of infection: direct and environmental transmission and the role of survivors, that delay the resurgence of viraemia, are essential to capture the population crash associated with the initial ASF epidemic and long-term persistence of ASF that regulates the host at low density. The long-term persistence of ASF makes the virus difficult to eradicate and increases the opportunity of infectious spread to neighbouring populations.

Recent studies have highlighted the potential role of chronically infected and survivor individuals in the transmission of ASF^14,39,41^. Eblé *et al*.^41^ showed that chronically (or sub-chronically) infected domestic pigs of the Netherlands ’86 strain of ASF (a low virulence strain) could transmit the infection through contact to susceptible pigs leading to acute infection. Also, Ståhl *et al*.^14^ suggests there are two types of individuals that may survive initial infection: (i) those that do not initially die of the disease but develop a persistent infection and can succumb to the disease and excrete virus in association with the resurgence of viraemia and (ii) those that show no clinical signs of infection, that can clear the infection and would not present prolonged virus excretion. The significance of these types is likely to vary between hosts and strains of the virus^14,41^. Our model study considers individuals that survive an initial infected and infectious stage and that can later revert to the infected class that can transmit the virus (type (i) in the Ståhl *et al*.^14^ definition of survivor pigs). In the absence of this survivor stage our results indicate that the disease is self-limiting and fades out after the initial epidemic, which is typical of high virulence infection^15^. The survivor stage therefore adds a delay that allows the infection to persist in the long-term.

While Ståhl *et al*.^14^ suggest that there is evidence for individuals that survive ASF infection they also question whether such survivors would play a role in the persistence of ASF. We therefore used the model to further explore ASF transmission mechanisms that could lead to the long-term persistence of ASF. In our model study survivor individuals cannot transmit the infection, whereas there is evidence that they can excrete the virus and therefore have the potential to transmit infection^14,41^. An extension of our model study, which also included infection transmission from the survivor class, showed that the rate of transmission must be low compared to that of acutely infected individuals (Fig. S21) but that its inclusion allowed the model to match the epidemiological criteria for ASF with a reduced overall density of survivor individuals (Fig. S22). There is also evidence of variability in the degradation of wild boar carcasses where in some cases skeletonisation can take several months and is dependent on factors such as insect activity, scavenger activity and weather conditions^42^. An extension of the model showed that the epidemiological criteria for ASF could not be satisfied for a range of carcass degradation rates in the absence of survivor individuals (representing an average degradation length from 1 week to 40 weeks). When carcass degradation is slow and environmental transmission is ‘high’, it is possible to satisfy our epidemiological criteria points (1) and (2) related to the drop in population density and timing of the peak outbreak of ASF but not the persistence of ASF in the long-term since ASF fades-out after the outbreak (Fig. S23). When carcass degradation is slow and environmental transmission is ‘low’, it is possible to satisfy point (3) of our epidemiological criteria related to the long-term persistence of ASF but the decrease in host density is slow and the peak in the outbreak of ASF occurs several years after the initial introduction of the infection. While we recognise that models are a simplified representation of the real world and that there is uncertainty in the criteria we use to define the epidemiological dynamics of ASF, our modelling results combined with recent empirical assessments^14,41^, suggest that a more detailed analysis of the role of survivor individuals in ASF epidemiology should be undertaken.

The results allow us to compare the epidemiology of ASF in scenarios that represent different regions or countries. For Estonia and other northern European regions, it is assumed that degradation of infected carcasses is slow^18^. Consequently, direct environmental transmission is the key infection mechanism that drives an epidemic outbreak and rapid population crash.

In Spain or other southern European countries, degradation of infected carcasses is likely to be faster due to elevated temperatures and the potential role of obligate scavengers. It was recently suggested that in northern Europe scavengers represent a minor risk factor for spreading ASF, but may contribute to reducing virus persistence^43^. Here, the model predicts that the epidemic outbreak will be less severe and the wild boar population loss from ASF infection will be reduced due to the high degradation rate of infected carcasses. Our results can inform the ongoing debate regarding vulture conservation^44^. We acknowledge that the role of scavengers and temperature on carcass degradation in Spain is uncertain and likely to vary across regions and habitat type. In open areas there is evidence that vultures can clean a carcass in a matter of hours^35^. If the degradation rate is suitably rapid (on average 1 day) then the infection cannot persist (Fig. S19). However, in covered or wooded habitats a carcass may go undetected and could degrade more slowly. As this degradation rate decreases, the results in Spain become more similar to those in Estonia with a predicted population crash of 85–95% following ASF introduction (Fig. S20). This suggests that there may be considerable local and regional variation in the impact of ASF. We do not include the impact of obligate scavengers in Estonia (since they are not present) but there may be an impact from the partial consumption of carcasses from other scavengers (such as wolves, birds or insects^42^). The impact of such activity would be similar to the impact of increased carcass removal through control.

It is noteworthy that in both Estonia and Spain the higher underlying host density and longer breeding season associated with supplementary feeding leads to a more pronounced epidemic outbreak and persistence of the disease in the long-term. This increased disease risk in supplementary fed populations fits with similar empirical findings regarding other viral and bacterial infections of wild boar^45,46^. Therefore, wild boar feeding should be limited as a means of ASF prevention, at least in open, unfenced areas.

Recent assessments on the spread and control of ASF advocate the use of hunting, culling and the active removal of carcasses as potential methods by which to control the infection^7^. Our study indicates that multiple control methods should be applied in parallel to eradicate ASF without eradicating the population. This will be of particular relevance in regions where wild boar hunting is an important industry that supports rural communities^47^. Furthermore, the control methods are more effective if implemented at the onset of infection (or prior to the arrival of infection in the case of culling) as they reduce the size of the infectious outbreak, thereby reducing the risk of spread to neighbouring populations. This supports the finding of Barongo *et al*.^6^, who tested the impact of the timing of bio-security measures on the control of ASF indicting that a rapid response was more effective.

The use of culling as a method to control infectious disease has had varying levels of success^48^,^49^. A recent theoretical paper has shown that in hosts challenged by highly virulent pathogens that do not confer long-lived immunity there is only a narrow gap between the thresholds in the culling rate that eradicate the disease or that eradicate the population^32^. ASF is highly virulent with little prospect of post infection recovery to immunity. Our model study indicates that in Estonia, culling alone is unlikely to eradicate the disease without eradicating the host population, whereas in Spain culling could potentially eradicate the disease without eradicating the host. The difference is that in Estonia infected carcasses remain in the environment, acting as a long-term source of infection and increasing the difficulty of eradicating the disease. We also consider the potential of disease control through the removal of infected carcasses. Carcass removal reduces density dependent environmental transmission and thereby reduces the population crash at the onset of infection. This allows an increased density of hosts to be supported in the long-term. For some scenarios however, the lower level of transmission combined with the increased density of hosts means the density of infected individuals can increase in response to control. In all cases carcass removal alone, instigated when the infection is detected, cannot eradicate the virus. When culling and carcass removal control methods are combined the model predicts that disease eradication can occur without the eradication of the host population. Here the increase in mortality due to culling is balanced by the reduction in transmission due to carcass removal and therefore a reduction in disease induced mortality at the population level^32^.

The threat posed by ASF to wild and domestic swine is significant^4,6^, and understanding the transmission dynamics is key for developing strategies to control ASF^7^. Our model study considers the dynamics and impacts of ASF control measures for different population scenarios that are representative of different geographical regions and different wild boar management strategies. Our findings have uncovered the role of different infection transmission routes in determining the epidemiological dynamics and in particular we suggest that a small proportion of survivor individuals that can subsequently revert to the infected class can play a key role in the long-term persistence of infection. The model results also suggest that ASF eradication requires a combination of control measures applied at the onset of (or prior to) detection of infection. This study highlights the role that mathematical modelling can play in understanding and developing management strategies to control important infectious diseases.

## Supplementary information

Supplementary Information.
