# Supplementary material for: Modelling the transmission and persistence of African swine fever in wild boar in contrasting European scenarios
Source: Sci Rep. 2020 Apr 3;10:5895. doi: 10.1038/s41598-020-62736-y (PMC7125206; doi:10.1038/s41598-020-62736-y)
Supplement: Supplementary file 1 — Supplementary Information. [file 41598_2020_62736_MOESM1_ESM.pdf]

Supplementary Information for:

Modelling the transmission and persistence of African swine fever in wild boar in contrasting European scenarios

Xander O'Neill[1], Andrew White[1,*], Francisco Ruiz-Fons[2], Christian Gortázar[2]

1 - Maxwell Institute for Mathematical Sciences, Department of Mathematics, Heriot-Watt University, Edinburgh, UK, EH14 4AS

2 - SaBio, Instituto de Investigación en Recursos Cinegéticos IREC (UCLM & CSIC), 13005 Ciudad Real, Spain

*Corresponding Author: A.R.White@hw.ac.uk

February 26, 2020

S1 The birth rate function, $a(t)$

We use either uni-modal or bi-modal birth functions, Figure S1, to represent the reproductive conditions when there is a natural population, Figure S1A, and a population with supplementary feeding, Figure S1B. In the former case each female adult is expected to produce 6 offspring with a peak of reproduction in March. As we assume a 50/50 male/female split, we assume each adult, on average, to produce 3 offspring. With additional feeding, we expect two peaks in reproduction, one in March and one in September, and an increase in the offspring per female to 12 individuals.

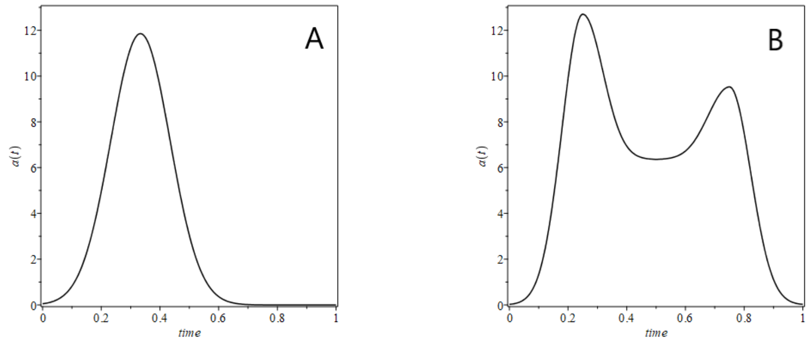

Figure S1: The average reproductive rate, $a(t)$, for adult wild boar over a period of 1 year, where January is given by $t = 0$, in natural conditions (A) and with supplementary feeding (B) respectively. Under natural conditions we assume each adult individual produces an average of 3 piglets per year, and with supplementary feeding an average of 6 piglets per year.

S2 Drivers for the epidemiological dynamics of African swine fever

We undertake a sensitivity analysis to find the transmission parameters and the proportion transitioning to the survivor state that satisfy the criteria outlined in section 2 of the main paper (Figure S2). In particular the epidemiological criteria are only satisfied for a limited range of transmission coefficients and proportions transitioning to survivors.

Density dependent environmental transmission is the key process driving the initial population crash. Model results when environmental transmission is excluded (Figure S3A) show no epidemic outbreak, and as a result no drop in population density and when environmental transmission is high (Figure S3C) the crash in population is severe and drops below the level defined by our epidemiological criteria.

Frequency dependent transmission and the progression and subsequent reversion from survivor to infected individuals allows the infection to be sustained at low density. Without these processes the disease is self-limiting and fades out after the epidemic (Figure S4, Figure S5). If frequency dependent transmission is high the population exhibits pathogen driven extinction (Figure S4C) and if a high proportion of infected individuals progress to the survivor phase the population does not exhibit such a marked crash.

In our model study survivor individuals revert back to the infected state after an average of six months, but there is uncertainty in this duration [34]. We use the model to show that the epidemiological criteria for ASF can be satisfied for a range of durations in the survivor class (from 2

months to 9 months, see Figure S6). In order to satisfy the epidemiological criteria when the length of time in the survivor class decreases, the proportion of individuals that survive the infection must increase, leading to an increase in the density of survivor individuals.

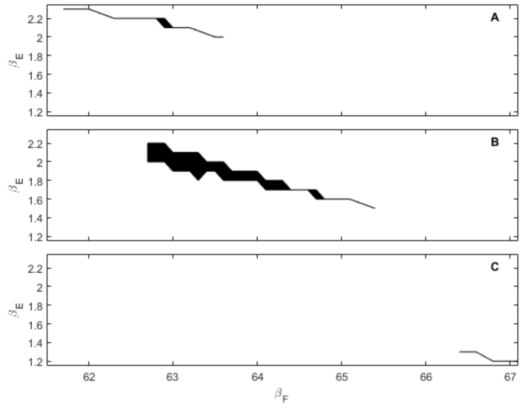

Figure S2: A selection of valid transmission coefficients satisfying the epidemiological criteria outlined in section 2 of the main paper for the model represented by equations (1) and for parameters that represent Estonia under natural conditions with no control measures. We also test the significance of when the infection is introduced and the results shown are for parameters that satisfy the epidemiological criteria when an outbreak may occur at any time of the year. The shaded regions indicate parameters that satisfy the epidemiological criteria for $\rho = 0.83$ in A; $\rho = 0.85$ in B; $\rho = 0.9$ in C. The other parameters used are outlined in section 2.1.

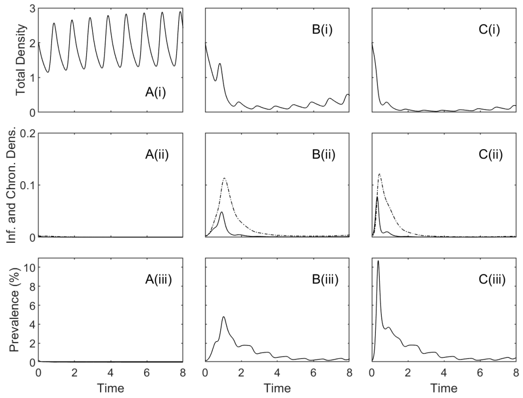

Figure S3: Model results for the scenario that represents Estonia under natural conditions for different environmental transmission coefficients: $\beta_E = 0$ (A), $\beta_E = 2$ (B) and $\beta_E = 6$ (C), with other parameters set to default values (in particular $\beta_F = 63$ and $\rho = 0.85$). Plots in (i) give the total densities, (ii) the infected (solid line) and survivor (dashed) densities and (iii) the prevalence. Other parameters are as discussed in section 2.1. When environmental transmission is low there is no population crash (A) and when it is high the crash is too severe (C).

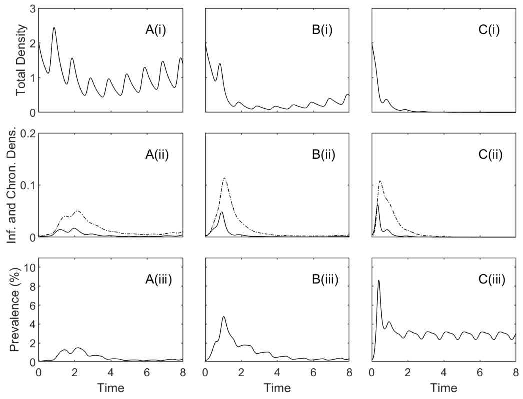

Figure S4: Model results for the scenario that represents Estonia under natural conditions for different frequency dependent transmission coefficients: $\beta_F = 50$ (A), $\beta_F = 63$ (B) and $\beta_F = 76$ (C), with other parameters set to default values (in particular $\beta_E = 2$ and $\rho = 0.85$). Plots in (i) give the total densities, (ii) the infected (solid line) and survivor (dashed) densities and (iii) the prevalence. Other parameters are as discussed in section 2.1. When frequency dependent transmission is low the prevalence following an outbreak is too low (A) and when it is high the population exhibits pathogen driven extinction (C).

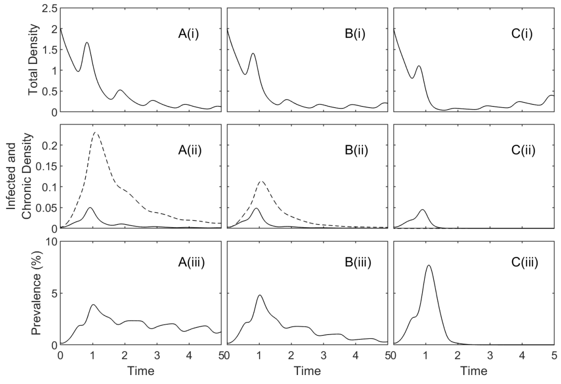

Figure S5: Model results for the scenario that represents Estonia under natural conditions for different values of $\rho = 0.7$ (A), $\rho = 0.85$ (B) and $\rho = 1$ (C), with other parameters set to default values (in particular $\beta_F = 63$ and $\beta_E = 2$). Plots in (i) give the total densities, (ii) the infected (solid line) and survivor (dashed) densities and (iii) the prevalence. Other parameters are as discussed in section 2.1. If the proportion that transition to the survivor state is high then the population crash is too slow (A) and if it is too low the disease fades out after the infectious outbreak (C).

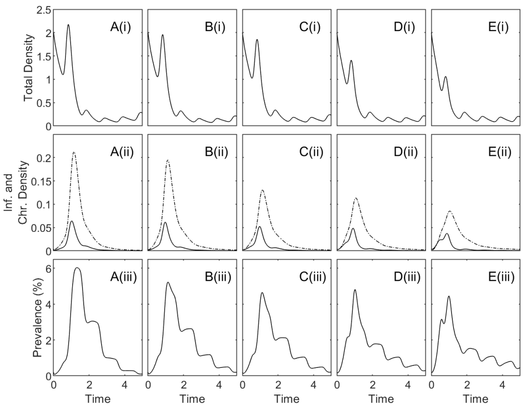

Figure S6: Population densities and prevalence over time for the model described by equations (1). Total densities are given in (i), infected (solid line) and survivor (dashed line) densities in (ii), with prevalence, defined as I/N, in (iii). Simulations were completed for the situation representative of Estonia under natural conditions for different values of the survivor reversion rate κ, with results for $\kappa = 12/2, 12/3, 12/4, 12/6$ and $12/9$ given in figures A, B, C, D and E respectively. This corresponds to an average time in the survivor class of $2, 3, 4, 6$ and 9 months in A, B, C, D and E respectively. The transmission parameters are (β_F, β_E, ρ) are given by $(40, 4, 0.61)$, $(50, 3.1, 0.7)$, $(58, 2.1, 0.8)$, $(63, 2, 0.85)$ and $(67.5, 1.7, 0.89)$ for figures $A - E$ respectively, so that the epidemiological criteria, described in section 2, are satisfied. All other parameters are as discussed in Figure 1.

S3 The impact of control in Estonia with supplemented feeding

Figures S7, S8, S9 & S10 explore the impact of culling and carcass removal on the epidemiological dynamics for the parameters representing the scenario in Estonia where wild boar receive supplementary feeding.

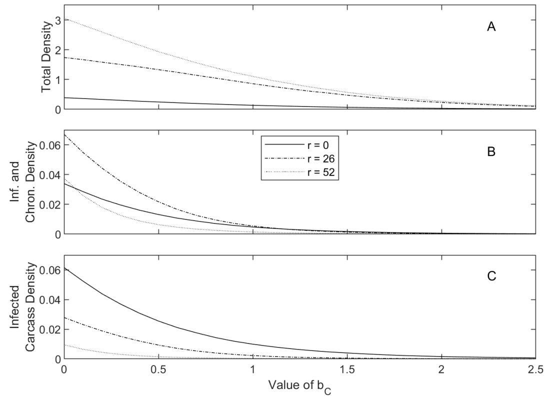

Figure S7: Population response to a varying culling intensity, b_C, with three different carcass removal rates $r = 0$ (solid line), $r = 26$ (dashed) and $r = 52$ (dotted), for the model represented by equations (1). The total density, N, is given in A, with infected and survivor density in B and carcass density in C. Results are shown for the scenario that represents Estonia with supplementary feeding (see Figure 1 for parameters) and show the average densities between the years 2 and 3 following disease introduction. Control measures were implemented as soon as the virus is first discovered, defined as the time when carcass levels first reach a density of 0.02.

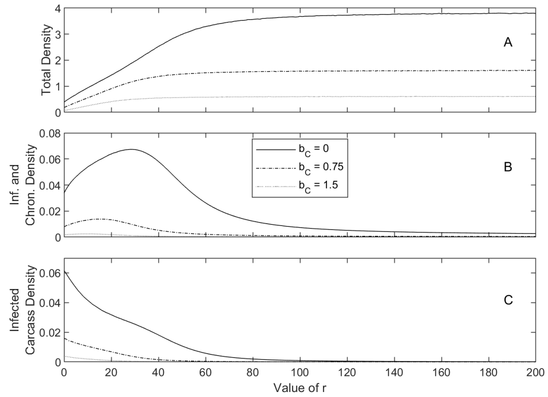

Figure S8: Population response to a varying carcass removal rate, r, with three different culling intensities $b_C = 0$ (solid line), $b_C = 0.75$ (dashed) and $b_C = 1.5$ (dotted), for the model represented by equations (1). The total density, N, is given in A, with infected and survivor density in B and carcass density in C. Results are shown for the scenario that represents Estonia with supplementary feeding (see Figure 1 for parameters) and show the average densities between the years 2 and 3 following disease introduction. Control measures were implemented as soon as the virus is first discovered, defined as the time when carcass levels first reach a density of 0.02.

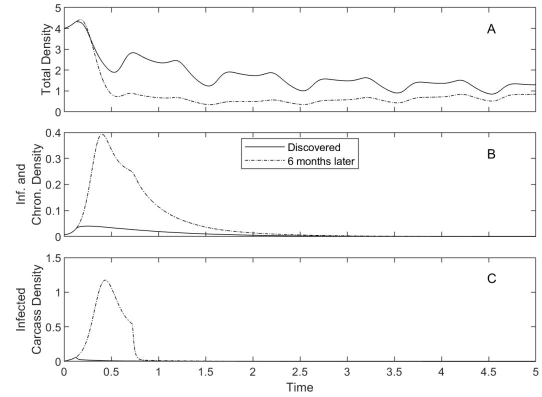

Figure S9: Population response to the combination of culling, at fixed rate $b_C = 0.75$, and carcass removal, at fixed rate $r = 52$, for the model represented by equations (1) and for parameters that represent the scenario of Estonia with supplementary feeding (see Figure 1 for parameters).The total density, N, is given in A, with infected and survivor densities in B and prevalence in C. The results are shown for two different control implementation times: when the virus is first discovered (solid line), and six months after the virus was discovered (dashed line).

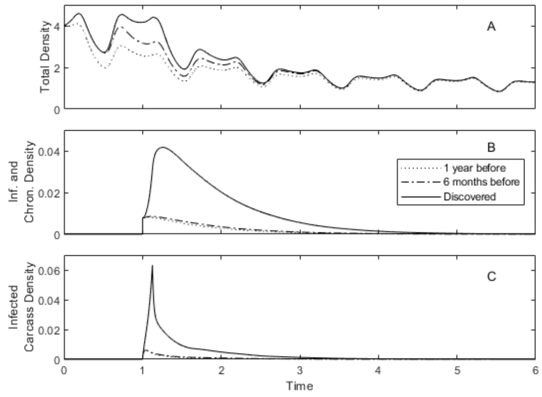

Figure S10: Population response to the combination of culling, at fixed rate $b_C = 0.75$, and carcass removal, at fixed rate $r = 52$, for the model represented by equations (1) and for parameters that represent the scenario of Estonia with supplementary feeding (see Figure 1 for parameters).The total density, N, is given in A, with infected and survivor densities in B and prevalence in C. The results are shown for three different control implementation times: one year before the onset of the virus (dotted line), six months before the onset of the virus (dashed line), and when the virus is first discovered (solid line).

S4 The impact of control in Spain under natural conditions

Figures S11, S12, S13 & S14 explore the impact of culling and carcass removal on the epidemiological dynamics for the parameters representing the scenario in Spain where wild boar do not receive supplementary feeding.

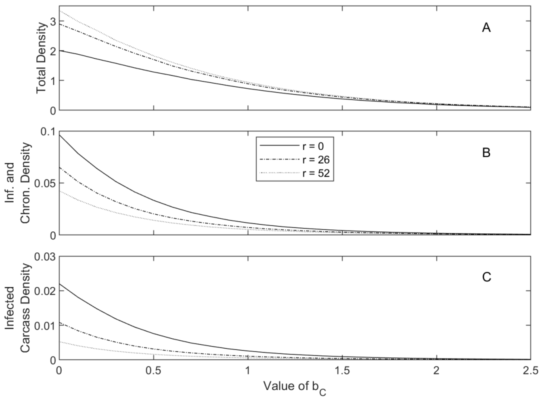

Figure S11: Population response to a varying culling intensity, b_C, with three different carcass removal rates $r = 0$ (solid line), $r = 26$ (dashed) and $r = 52$ (dotted), for the model represented by equations (1). The total density, N, is given in A, with infected and survivor density in B and carcass density in C. Results are shown for the scenario that represents Spain under natural conditions (see Figure 1 for parameters) and show the average densities between the years 2 and 3 following disease introduction. Control measures were implemented as soon as the virus is first discovered, defined as the time when carcass levels first reach a density of 0.02.

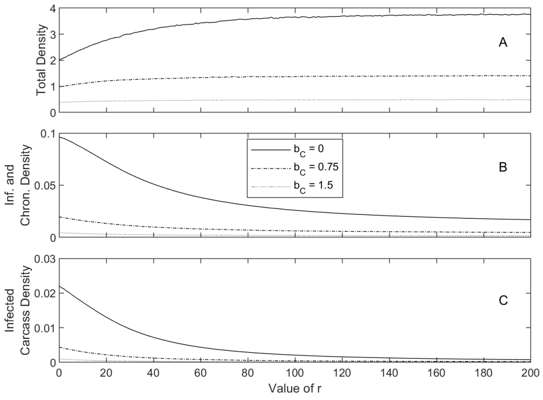

Figure S12: Population response to a varying carcass removal rate, r, with three different culling intensities $b_C = 0$ (solid line), $b_C = 0.75$ (dashed) and $b_C = 1.5$ (dotted), for the model represented by equations (1). The total density, N, is given in A, with infected and survivor density in B and carcass density in C. Results are shown for the scenario that represents Spain under natural conditions (see Figure 1 for parameters) and show the average densities between the years 2 and 3 following disease introduction. Control measures were implemented as soon as the virus is first discovered, defined as the time when carcass levels first reach a density of 0.02.

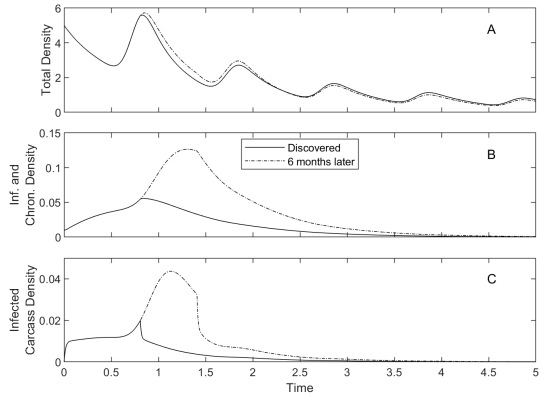

Figure S13: Population response to the combination of culling, at fixed rate $b_C = 0.75$, and carcass removal, at fixed rate $r = 52$, for the model represented by equations (1) and for parameters that represent the scenario in Spain under natural conditions (see Figure 1 for parameters).The total density, N, is given in A, with infected and survivor densities in B and prevalence in C. The results are shown for two different control implementation times: when the virus is first discovered (solid line), and six months after the virus was discovered (dashed line).

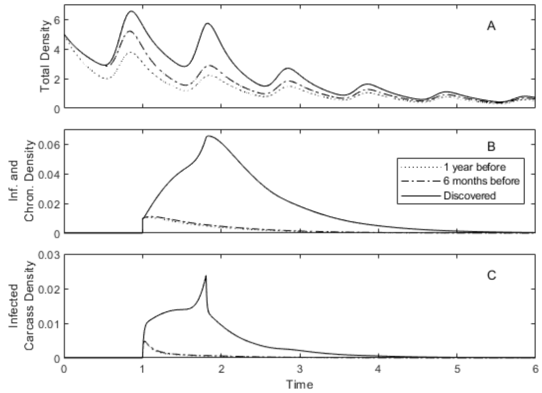

Figure S14: Population response to the combination of culling, at fixed rate $b_C = 0.75$, and carcass removal, at fixed rate $r = 52$, for the model represented by equations (1) and for parameters that represent the scenario in Spain under natural conditions (see Figure 1 for parameters).The total density, N, is given in A, with infected and survivor densities in B and prevalence in C. The results are shown for three different control implementation times: one year before the onset of the virus (dotted line), six months before the onset of the virus (dashed line), and when the virus is first discovered (solid line).

S5 The impact of control in Spain with supplemented feeding

Figures S15, S16, S17 & S18 explore the impact of culling and carcass removal on the epidemiological dynamics for the parameters representing the scenario in Spain where wild boar receive supplementary feeding.

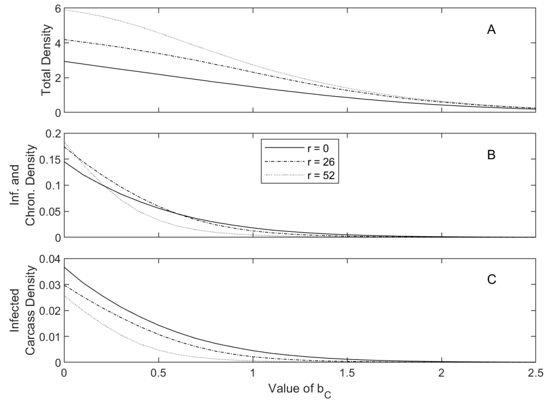

Figure S15: Population response to a varying culling intensity, b_C, with three different carcass removal rates $r = 0$ (solid line), $r = 26$ (dashed) and $r = 52$ (dotted), for the model represented by equations (1). The total density, N, is given in A, with infected and survivor density in B and carcass density in C. Results are shown for the scenario that represents Spain with supplementary feeding (see Figure 1 for parameters) and show the average densities between the years 2 and 3 following disease introduction. Control measures were implemented as soon as the virus is first discovered, defined as the time when carcass levels first reach a density of 0.02.

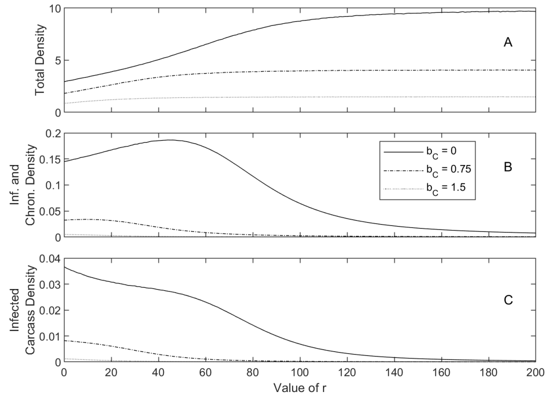

Figure S16: Population response to a varying carcass removal rate, r, with three different culling intensities $b_C = 0$ (solid line), $b_C = 0.75$ (dashed) and $b_C = 1.5$ (dotted), for the model represented by equations (1). The total density, N, is given in A, with infected and survivor density in B and carcass density in C. Results are shown for the scenario that represents Spain with supplementary feeding (see Figure 1 for parameters) and show the average densities between the years 2 and 3 following disease introduction. Control measures were implemented as soon as the virus is first discovered, defined as the time when carcass levels first reach a density of 0.02.

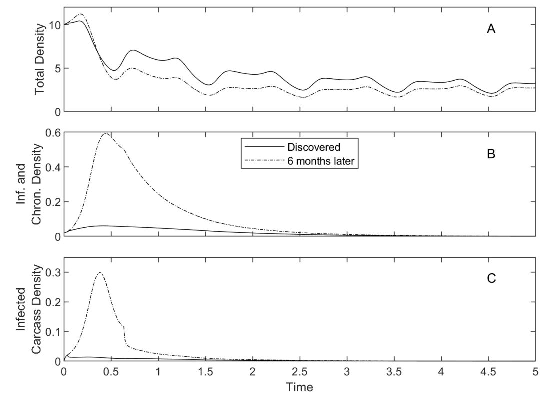

Figure S17: Population response to the combination of culling, at fixed rate $b_C = 0.75$, and carcass removal, at fixed rate $r = 52$, for the model represented by equations (1) and for parameters that represent the scenario of Spain with supplementary feeding (see Figure 1 for parameters).The total density, N, is given in A, with infected and survivor densities in B and prevalence in C. The results are shown for two different control implementation times: when the virus is first discovered (solid line), and six months after the virus was discovered (dashed line).

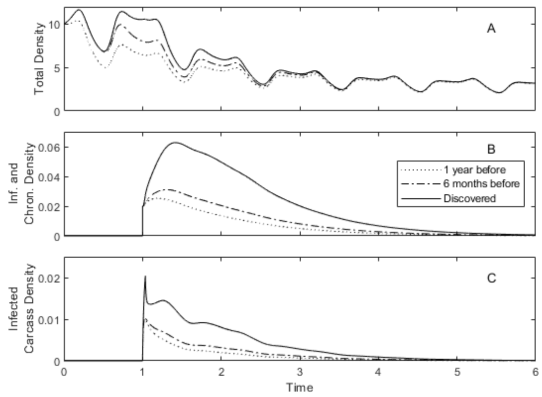

Figure S18: Population response to the combination of culling, at fixed rate $b_C = 0.75$, and carcass removal, at fixed rate $r = 52$, for the model represented by equations (1) and for parameters that represent the scenario of Spain with supplementary feeding (see Figure 1 for parameters).The total density, N, is given in A, with infected and survivor densities in B and prevalence in C. The results are shown for three different control implementation times: one year before the onset of the virus (dotted line), six months before the onset of the virus (dashed line), and when the virus is first discovered (solid line).

S6 The impact of a varying degradation rate in Spain under natural conditions

Figures S19 and S20 explore the impact of a varying degradation rate, d, for the parameters and scenario representative of Spain under natural conditions in the absence of control.

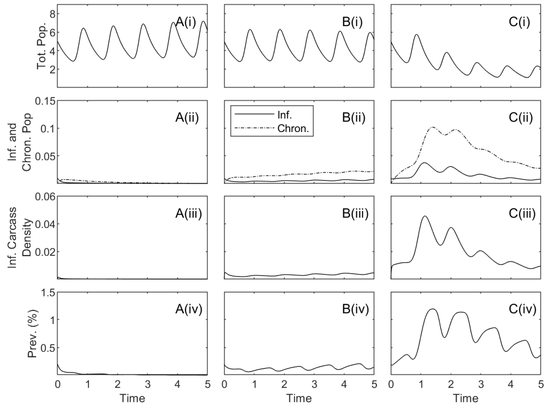

Figure S19: Model simulations for the scenario representative of Spain under natural conditions with no control measures (see Figure 1 for parameters). The degradation rate varies from 1 day ($d = 365$), A, to 1/2 a week ($d = 104$), B, and to 1 week ($d = 52$), C, with C being our default. The total density, N, is given in (i), with infected (solid line) and survivor densities (dashed line) in (ii), infected carcass densities in (iii) and prevalence in (iv).

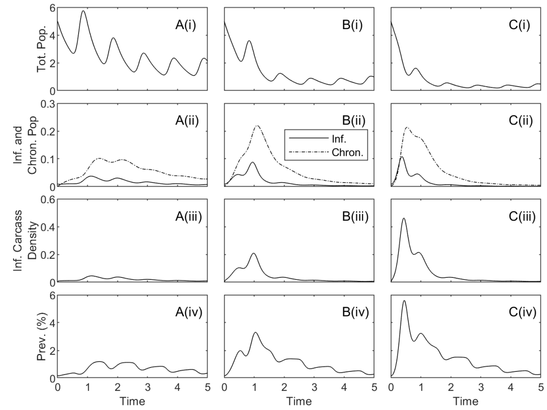

Figure S20: Model simulations for the scenario representative of Spain under natural conditions with no control measures (see Figure 1 for parameters). The degradation rate varies from 1 week ($d = 52$), A, to 2 weeks ($d = 26$), B, and to 4 weeks ($d = 13$), C, with A being our default. The total density, N, is given in (i), with infected (solid line) and survivor densities (dashed line) in (ii), infected carcass densities in (iii) and prevalence in (iv).

S7 Transmission from survivor individuals

Figures S21 and S22 explore the range of parameters which satisfy the epidemiological criteria for ASF (section 2) when survivor individuals can also transmit the infection. We assume that survivor individuals transmit at rate $\beta_C = F\beta_F$ which is a fraction, F, of the rate of transmission from infected individuals. For both our default parameters and those which consider a reduced level of β_F the rate of transmission from survivor individuals needs to be low for the dynamics to satisfy the epidemiological criteria of ASF (Figure S21). The inclusion of transmission from survivor individuals allowed the model to match the epidemiological criteria for ASF with a reduced overall density of survivors (Figure S22). Note, if the transmission rate from survivor individuals is similar to that of acutely infected individuals then it leads to population extinction.

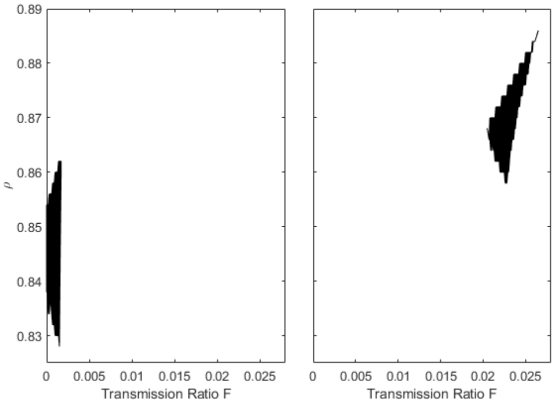

Figure S21: A selection of valid transmission coefficients satisfying the epidemiological criteria outlined in section 2 of the main paper for the model that represents Estonia under natural conditions and which additionally includes transmission from survivor individuals with transmission rate $\beta_C = F\beta_F$. Parameters are as in section 2.1 with $\beta_F = 63$ in (A) and $\beta_F = 56$ in (B).

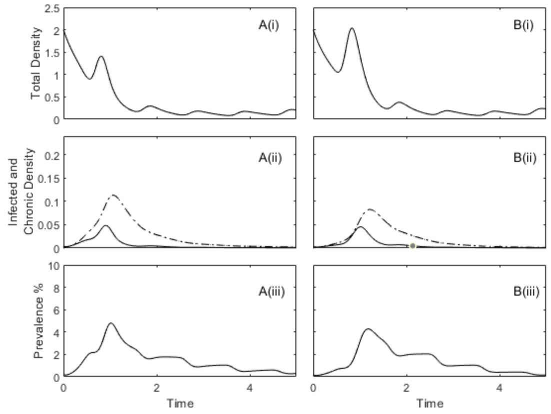

Figure S22: Population densities and prevalence over time for the model representative of Estonia under natural conditions. In (A) the model is described by equations (1) with parameters as outlined in section 2.1 and in (B) the model additionally includes transmission from survivor individuals with transmission rate $\beta_C = F\beta_F$ and parameters as outlined in section 2.1 except for $\beta_F = 56$ and $F = 0.025$. The total density, N, is given in (i), with infected (solid line) and survivor densities (dashed line) in (ii), and prevalence in (iii)

S8 Varying Carcass Degradation Rates without a Survivor Population

We used the model to test whether the epidemiological criteria for ASF could be satisfied for a range of carcass degradation rates (representing an average degradation time from 1 week to 40 weeks) in the absence of a survivor class. However, the epidemiological criteria could not be satisfied. When carcass degradation is slow and environmental transmission is 'high', it is possible to satisfy our epidemiological criteria points (1) and (2) related to the drop in population density and timing of the peak outbreak of ASF but not the persistence of ASF in the long-term since ASF fades-out after the outbreak (Figure S23). This result only occurred for degradation lengths of less than 26 weeks. When carcass degradation is slow and environmental transmission is 'low', it is possible to satisfy point (3) of the epidemiological criteria related to the long-term persistence of ASF but the decrease in host density is slow and the peak in the outbreak of ASF occurs several years after the initial introduction of the infection. This result only worked for degradation lengths of more than 37 weeks.

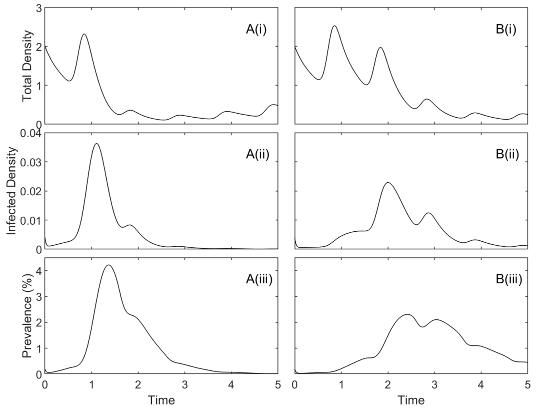

Figure S23: Population densities and prevalence over time for the model described by equations (1), with the total densities given in (i), infected density in (ii), and prevalence, defined as I/N, in (iii). Simulations were run for the situation representative of Estonia under natural conditions but without the presence of a survivor class ($\rho = 1$). Note, in the absence of a survivor class the three epidemiological criteria defined in section 2 cannot be satisfied. For plots in (A) we use parameters ($\beta_F = 42, \beta_E = 2, d = 20$) satisfying the first two elements of the epidemiological criteria but that could not satisfy the third and in (B) we use parameters ($\beta_F = 42, \beta_E = 1, d = 40$) that could satisfy the third element in the epidemiological criteria but not the first two. All other parameters are as in Figure 1.
